# What are Digital Public Health Interventions? First Steps Toward a Definition and an Intervention Classification Framework

**DOI:** 10.2196/31921

**Published:** 2022-06-28

**Authors:** Julian Wienert, Tina Jahnel, Laura Maaß

**Affiliations:** 1 Research Cluster Evaluation Leibniz ScienceCampus Bremen Digital Public Health Bremen Germany; 2 Social Epidemiology Prevention and Evaluation Leibniz Institute for Prevention Research and Epidemiology – BIPS Bremen Germany; 3 Health Psychology Social Sciences IU International University for Applied Sciences Bad Reichenhall Germany; 4 Department of Health Services Research Institute for Public Health and Nursing University of Bremen Bremen Germany; 5 Health, Long-Term Care and Pensions Research Center on Inequality and Social Policy (SOCIUM) Bremen Germany

**Keywords:** digital health, digital Public Health, digital public health interventions, digital health technologies, mHealth, eHealth, participatory approach, framework, mobile phone

## Abstract

Digital public health is an emerging field in population-based research and practice. The fast development of digital technologies provides a fundamentally new understanding of improving public health by using digitalization, especially in prevention and health promotion. The first step toward a better understanding of digital public health is to conceptualize the subject of the assessment by defining what digital public health interventions are. This is important, as one cannot evaluate tools if one does not know what precisely an intervention in this field can be. Therefore, this study aims to provide the first definition of digital public health interventions. We will merge leading models for public health functions by the World Health Organization, a framework for digital health technologies by the National Institute for Health and Care Excellence, and a user-centered approach to intervention development. Together, they provide an overview of the functions and areas of use for digital public health interventions. Nevertheless, one must keep in mind that public health functions can differ among different health care systems, limiting our new framework’s universal validity. We conclude that a digital public health intervention should address essential public health functions through digital means. Furthermore, it should include members of the target group in the development process to improve social acceptance and achieve a population health impact.

## Introduction

### Background

As digitization plays a large role in an increasing number of health systems, digital public health is an emerging field for population-based research and practice. The fast development of both hardware- and software-based digital technologies provides a fundamentally new understanding of improving public health, which can be achieved through digitalization, especially in prevention and health promotion. For example, digital technologies may improve physical activity levels, dietary intake, posture, and mental well-being via sensors and apps [1]. Technological innovations in apps for tracking health-related behavior, monitoring potential health risks, and communication and interaction have rapidly changed many aspects of public health [2]. However, not all of these interventions might achieve a health impact at a population level by displaying effectiveness in randomized clinical trials and efficacy under quasi-experimental real-world circumstances.

Although there is a need for evaluation methods that address the many challenges that arise with digitization (eg, fast-paced development), it is challenging to assess digital public health interventions as these may span from population health surveillance to the prevention of specific diseases, and they develop faster than analog interventions [3]. Moreover, companies and institutions often develop digital tools based on market evaluations, expected profits, and technological possibilities but not based on the public’s needs and preferences. To improve digital public health interventions’ effectiveness and efficacy, we first need to understand what they entail and how they are defined. However, to our knowledge, no definition for digital public health interventions exists to date. Only by doing that will we gather meaningful, valid, and reliable results on their effectiveness and efficiency. Thus, the aim of this viewpoint is to offer a definition for digital public health interventions.

Before defining digital public health interventions, we need to explain the differences between eHealth, mobile health (mHealth), digital health, and digital public health. This is necessary to highlight the differences between digital health interventions (DHIs) and digital public health interventions. Following this, we will build a framework that might help to identify, structure, and classify digital public health interventions. Our definition and framework will rely on existing approaches from public health [4], digital health [5], and user-centered design [6]. The first section of building the framework will explain why we chose the selected models; we decided to use the Essential Public Health Functions (EPHFs) by the World Health Organization (WHO) for the public health level [4], the updated version of the *Evidence Standards Framework for Digital Health Technologies* by the National Institute for Health and Care Excellence (NICE) [5], and the *Participatory Health Research* approach by Wright [6]. After clarifying the reasons for choosing named approaches, we will explain each model and how they are related to digital public health and may be used for digital public health purposes. After setting the theoretical background for our definition, we will illustrate our findings using the German Corona App as an example to validate our digital public health intervention criteria. We will conclude with a definition for digital public health interventions and use this to propose a digital public health intervention classification framework (Multimedia Appendix 1).

### Differences Among eHealth, mHealth, Digital Health, and Digital Public Health

**Figure 1:**
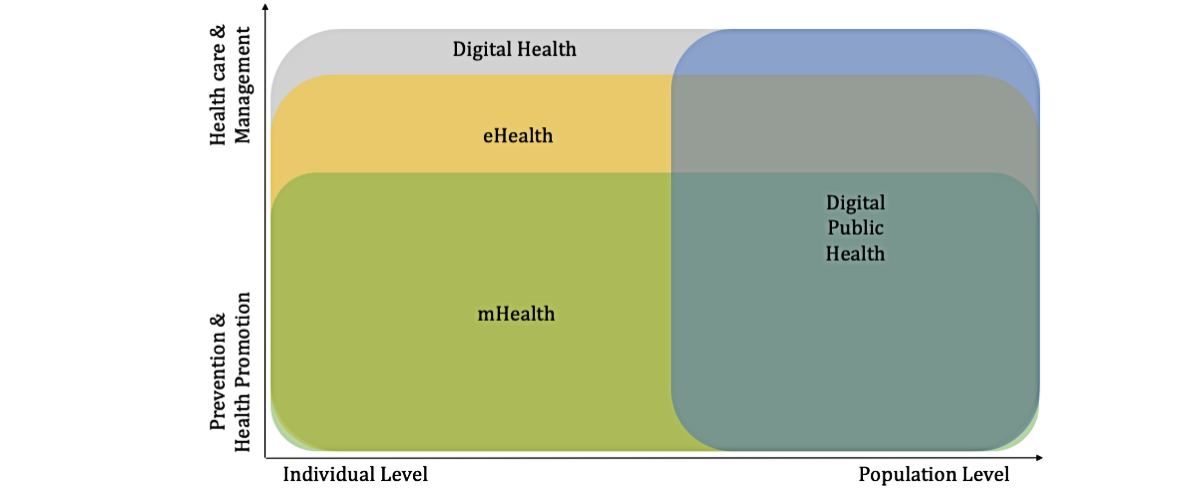
Core field of action and target group level of mHealth, eHealth, digital health, and digital public health [7-15]. mHealth: mobile health.

Terms such as eHealth, mHealth, or digital health are used in the context of the digitization of public health. Since 2019, few papers have also referred to the term digital public health. Given the multitude of terms and definitions in digital health, it is essential to understand the considerable heterogeneity of how such terms interrelate with each other and where digital public health might find its place in the terminological canon of digital health. Therefore, the following section will define the named terms and summarize their core fields of action and the target group’s level, as seen in Figure 1.

An article on *eHealth* concepts based on an extensive literature search [7] confirms the lack of consensus on the meaning of *eHealth* as possibly the first word in this field. A 2005 study found 51 different definitions of *eHealth* [8]. This lack of consensus highlights the importance of a shared understanding of terms. More recent studies emphasize that because of its immense dynamics, the field of *eHealth* is challenging to define [9]. Most definitions share the use of information and communication technologies (eg, the internet) for health topics. Their focus mostly lies on delivering health services rather than health promotion and disease prevention [10-12]. Some definitions also highlight the importance of user-centered approaches for facilitating health services in *eHealth* [10,12]. The word *mHealth* aims more directly at a particular technology, namely smartphones and mobile sensors, in their health significance. Thus, *mHealth* is defined more precisely overall, although different technologies are used here [13]. As a part of *eHealth*, the focus of *mHealth* lies on wireless and mobile technologies and their use in enhancing health-related science, treatment, and ultimately health status [9].

Fatehi et al [14] stated that in 2020, there were >90 different definitions for *digital health*. They concluded that *digital health* includes eHealth, mHealth, self-tracking, wearable devices, artificial intelligence, and information systems in health care, focusing on health and not technology. *Digital health* focuses on the health of individuals (eg, patients) to improve health care with technology [14]. This is where *digital health* and digital public health differ, as digital public health aims to improve health and well-being at the population level. Nevertheless, digital public health uses the same technologies that are also used to improve individual health care; however, its purposes change. A recent publication by Zeeb et al [15] provides the first overview of what digital public health might be. It serves as a starting point for developing a better understanding of digital public health interventions by providing a short introduction to central terms. Thus*,* following the article by Zeeb et al [15], the authors propose the following definition for digital public health in distinction to the other abovementioned fields and outline for which fields they see it relevant (own translation from German):

DiPH [...] focuses on the development, application, and knowledge interest on Public Health and thus on prevention, health promotion, and the related basic sciences such as epidemiology. Primary clinical and individual patient-related aspects are not in the foreground, unlike, for example, telemedicine with its concrete application in an individual treatment and care context. However, it should be noted that the term DiPH has not yet prevailed over others such as eHealth and mHealth. Also, this is hard to expect given the diversity and dynamics of the terms used to date. Where, however, the focus of digitization and health is on population, prevention, and health promotion, including a conscious analysis of health inequalities, DiPH can offer a clearer classification than some other terms in this field.

Although the definition by Zeeb et al [15] serves as a good starting point for the discussion, it mainly focuses on the primary level of prevention (ie, preventing a disease or injury before it occurs). Although public health, in its essence, comprises 3 levels of prevention, according to this definition, secondary (eg, reducing the impact of a disease or injury after it occurred) and tertiary preventions (eg, rehabilitation) are not explicitly mentioned by Zeeb et al [15] as part of digital public health. The central challenge of defining digital public health is the integration of digital development and technologies into public health concepts and use them to achieve public health goals rather than redefining and reconceptualizing public health in the face of technological advancements [5,15].

To develop a first working definition and classification framework for digital public health interventions, established models for both digital health [5] and public health [4] were assessed to combine aspects of these models to develop a more holistic definition of digital public health interventions. Here we suggest that digital public health, as a complex intervention, should be viewed from different perspectives. As such, our proposed classification is a combination of the elements of already existing models. Specifically, the EPHF by the WHO provided us with an overview of the necessary core functions of public health, which might also be addressed by digital means. Our definition will explicitly include the area of health promotion (ie, focusing on health resources) and all three levels of prevention (ie, primary prevention to reduce the risk of disease development, secondary prevention as screening and early diagnosis, and tertiary prevention for rehabilitation), as all these levels are included in the EPHF by the WHO [4]. We will then link our definition to the participation approach: a user-centered model for the development of digital interventions to increase the acceptance of digital public health interventions among target groups [6]. This will create a framework that follows the concepts of public health (goals).

### Choice of Included Models

Following a narrative approach and based on the authors’ expertise and experience in the field of Public Health, three layers were identified: (1) overview, which is a larger operational layer where the central functions of digital public health are mapped; (2) structure, which is a layer that focuses on structuring digital public health activities (eg, by functions); and (3) improvement, which is a layer that specifically includes the individual perspective in the development and use of digital public health interventions. For each layer, a framework was identified based on the author’s expertise and previous experience with the frameworks and a nonsystematic literature search for alternative frameworks. The frameworks included are as follows: the EPHF by the WHO, which offers a macro view of public health topics [4]; the *Evidence Standards Framework for Digital Health Technologies* by the NICE, which categorizes DHIs for the UK setting [5]; and the *Steps of Participation Approach*, which was suggested by Wright [6].

**Figure 2:**
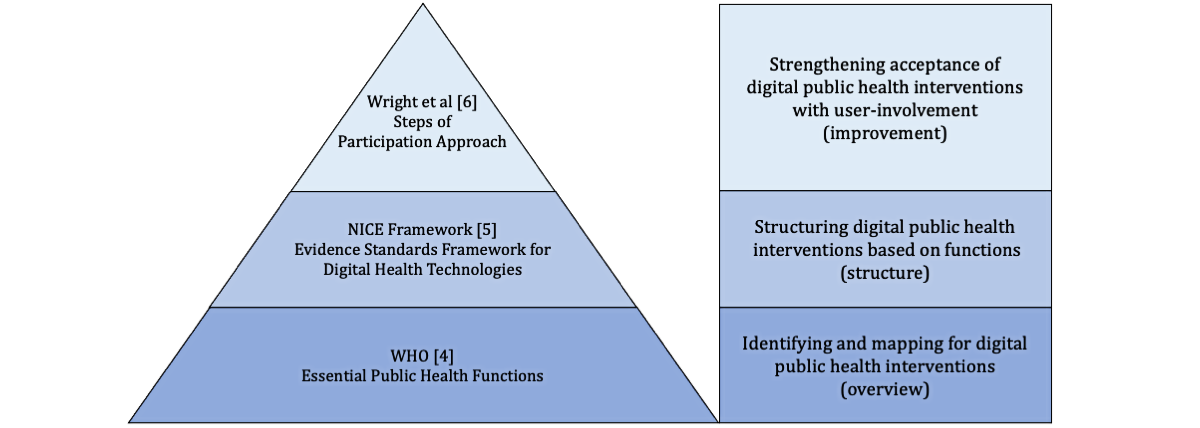
Conceptual pyramid for a framework of digital public health interventions [4-6]. NICE: National Institute for Health and Care Excellence; WHO: World Health Organization.

Together, the EPHF and the NICE framework will build the base of mapping and structuring digital public health interventions. We then use the *Steps of Participation Approach* suggested by Wright [6]. This is a user-centered approach for intervention development that aims to increase acceptance within the target group. The approach provides well-described and clear-cut categories for target group involvement—participation and nonparticipation alike. Together, with all 3 models aligned, a conceptual pyramid for digital public health intervention classification is formed (Figure 2).

### EPHFs by the WHO

A way of addressing public health goals to affect population health is using *the EPHF* [4]. Following the WHO report *EPHFs, health systems and health security: developing conceptual clarity and a WHO road map for action,* these functions can be separated into cross-cutting, horizontal functions, roughly based on the building blocks approach to health systems, and service-based, vertical functions comprising the traditional public health services provided by modern health systems [4]. Although there is no precise definition for each part, as they depend on each health care system or region, the WHO report identified some significant categories that most EPHF share (Textbox 1).

**Essential public health functions according to the World Health Organization [4].**

**Essential public health functions**

**Horizontal functions**

- Governance (eg, public health management, policy, and planning or quality assurance in health services)
- Financing: establishment of sustainable organizational structures, institutional capacity, and policy making
- Human resources: development and management of human resources
- Health information systems: population health surveillance and monitoring
- Research: development of a national public health research agenda, allocation of resources for research, integration of research activities into public health, capacity building for innovation, and dissemination to translate research findings into policy and practice
- Social participation and health communication: social participation, community partnership, community engagement and/or (digital) public health communication, and design public health services around people’s needs

**Vertical functions**

- Health protection: regulations and legal protections (for workers, patients, consumers, and the environment)
- Health promotion: community and social participation, intersectoral collaboration, measures to address behavioral risk factors (tobacco, alcohol, diet, and physical activity), and the social determinants of health and health education
- Disease prevention: services provided within the health care system and targeting communicable diseases
- Health care: With specific functions for quality assurance and access, universal health coverage is a defining feature depending on the World Health Organization region or country (ie, the European region emphatically excludes most health care services from the public health remit because of strong roots in the principles of universal access as opposed to the United States or Western Pacific regions).
- Preparedness for public health emergencies: encompass any sudden, large-scale, negative impact on public health arising from outbreaks, natural disasters, severe weather events, migratory flows, accidents, terrorism, or other environmental or human causes
- Other vertical functions: A wide variety of specific vertical functions are given importance in different countries. In part, this reflects an overall approach to developing frameworks that list essential services rather than broader functions per se. At the same time, the vertical positions were chosen to reflect national priorities.

These are just a few examples of fields for action in both (analog) public health interventions and digital public health interventions. However, we could not apply all EPHF to every setting. They depend strongly on specific health care systems, which differ among countries. In general, the WHO regards public health interventions primarily as an effort or policy that attempts to improve mental, social, and physical health at the population level by including and addressing EPHF [4].

Analogous to public health interventions, digital public health interventions have the potential to include and address horizontal as well as vertical functions. The governmental regulation of mHealth apps as medical devices with the possibility of reimbursement, as started in Germany in December 2020 [16], may be one way of applying horizontal EPHF of governance to digital public health. Various countries have also developed proximity-tracing apps as tools for population health surveillance and monitoring during the SARS-CoV-2 pandemic [17,18]. The last example of applying the horizontal EPHF to digital public health is the digitization of health care systems in total. This leads to a redesign around people’s needs and expectations in, for instance, web-based consultation services or telemedicine for people in rural areas who do not have access to health care professionals [19]. As for vertical public health functions, apps and wearables for self-monitoring, step counting, and fitness tracking can serve as examples of vertical digital public health functions. Their goal is to promote health and a healthy lifestyle [20,21].

### Level of Interaction: NICE Framework

Applying EPHF as a cornerstone for the identification and mapping of digital public health interventions provides an initial overview of the field of digital public health. The next step is to further structure such interventions based on their functional classification proposed by NICE’s *Evidence Standards Framework for Digital Health Technologies* [5]. This applied framework describes the types and levels of evidence needed to show the effectiveness and expected economic impact of a DHI. Various publications have used this framework for their digital health technology assessment, which confirmed our resolution that this framework is not only well-known but also well-used in the scientific field of digital health [22-24]. The NICE framework aims to establish standardized criteria that can assess DHIs by providing a functional classification and stratification into evidence tiers. This separation illustrates the main functions of the types of interventions that we expect to be the most widely developed (Textbox 2).

**Functional classification and stratification into evidence tiers [5].**

**Stratification into evidence tiers and functional classification**

**Evidence tier C: interventions**

- Preventive behavior change: address public health issues (eg, smoking, eating, alcohol, sexual health, sleeping, and exercise)
- Self-manage: allows people to self-manage a specific condition; may include behavior change techniques
- Treat: provides treatment and guides treatment
- Active monitoring: tracking patient location, using wearables to measure, record, and/or transmit data about a specific condition
- Calculate: a calculator that affects treatment, diagnosis, or care
- Diagnose: diagnose a specific condition; guides diagnosis

**Evidence tier B: understanding and communicating**

- Inform: provides information (about a condition or general health and lifestyle), resources, or activities to the public, patients, or clinicians
- Simple monitoring: includes general health monitoring using fitness wearables and simple symptom diaries
- Communicate: allows 2-way communication among citizens, patients, or health care professionals

**Evidence tier A: system impact**

- System service: digital health interventions with no measurable patient outcomes but which provide services to the health and social care system

The abovementioned evidence tiers serve as concrete examples in digital health for the EPHF. For instance, level A refers to the vertical EPHF *health care* (digital public health tools in this field could be electronic health records). In contrast, the 3 functions in evidence tier B belong to the horizontal public health function *social participation and health communication*. Recent examples for tier B are proximity-tracing apps (sometimes called contact-tracing apps), which various countries use in epidemic or pandemic outbreaks such as SARS-CoV-2. Such apps usually display level 2 functions (ie, informing, simple monitoring, and communication), which mirrors the underlying EPHF, including disease prevention and health information systems as underlying EPHF. The first 3 functions within evidence tier C serve as a digital example for the vertical functions of *health promotion* as well as *disease prevention* (such as mobile apps on prescription [16]). Finally, the last 3 functions in tier C, although focusing more on the medical and individual level than the other tiers and functions, can be seen as a part of *health promotion* and *disease prevention*. Unlike the first 3 functions in tier C, which focus more on the primary prevention area, the last 3 functions are more closely linked to secondary and tertiary prevention. Specifically, the functions of DHIs in tier C include the early diagnosis of specific conditions and rehabilitation and healing, which improves the user’s health (for instance, a national telemedicine service [19]).

As seen, there is an interrelation between the NICE framework and EPHF, supporting the argument that digital public health interventions can address EPHF. The critical part here is that the NICE framework, unlike the WHO EPHF, provides a structure for the degree of complexity (ie, level of interaction) based on the user’s risk. Following the understanding of complex and multicomponent interventions that act and interact on different levels, benefits, and acceptance of digital public health interventions, depending on the users of such interventions and their specific perspectives. Any digital public health intervention can ultimately fail if the population does not accept or use it. Thus, it is essential to involve target groups in the development of these interventions. We propose a participatory and user-centered approach for intervention development as the third cornerstone of digital public health interventions.

### User-Centered Approach in Intervention Development

Hochmuth et al [25] advocated that complex and multicomponent interventions require a user-oriented intervention design because of the varying intricacies of such interventions. This intricacy can be based on the following:

- Interactions between technological components (eg, sensors for data acquisition)
- Different requirements for users in the implementation of the intervention (eg, knowledge of data security)
- Involvement of other groups or organizational levels (eg, patients or researchers)
- Degree of adaptation or flexibility of the intervention (eg, further agile development through software updates) [3,25].

To follow a user-centered approach, developers must integrate the users (ie, the target group) in the development process. A way of structuring the involvement of users is the *Steps of Participation Approach* suggested by Wright [6]. This model describes the user’s nonparticipation and involvement in the research process. It further differentiates among 9 stages, ranging from instrumentalization to self-organization. The 9 stages provide a hierarchical order not only for participation but also for the nonparticipation of target groups in the development of public health interventions. Although it includes 9 stages, only the last 4 include real participation, according to Wright [6], as the first 2 have no target group members involved in the development process. Steps 3 to 5 are the precursors for participation. As stated by this approach, one can only speak of participation in only those areas where people have the power to participate in the decision-making processes [26]. The 9 significant steps based on Wright [6] are described in the following sections. As shown in Table 1, the difference among the 3 groups of nonparticipation is that the first 2 steps completely exclude the target group. Although grades 3 to 5 recognize the target group as advisers, they do not include them in the decision-making process, which occurs in steps 6 to 9 (Table 1). The chance to successfully roll out and implement a digital public health intervention increases as the development process includes the target group [27]. Therefore, a user-oriented way of developing digital public health interventions to increase acceptance and use of such interventions should be a goal of digital public health.

**Figure 3:**
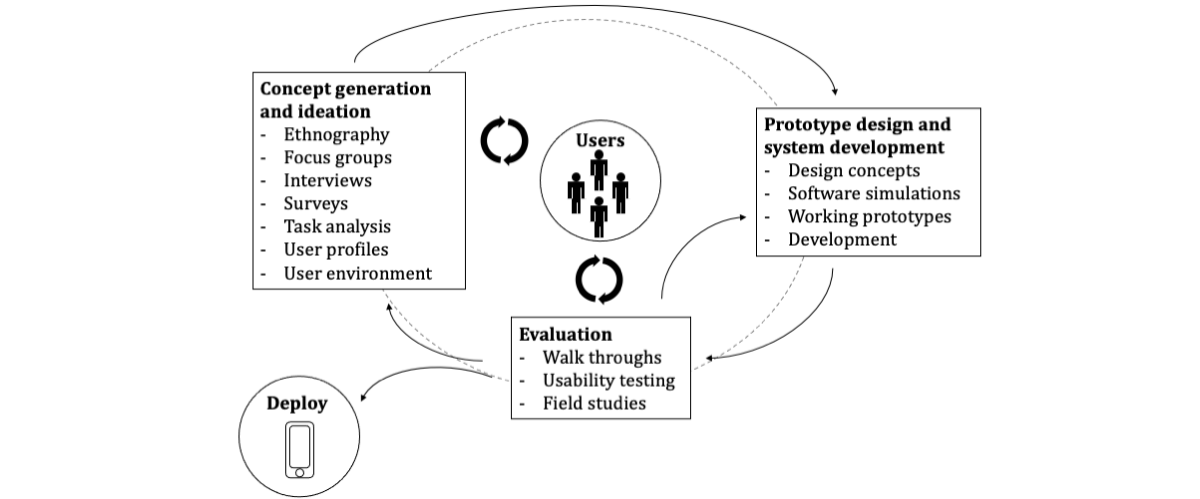
Schematic representation of the user-centered design process [28].

A way of including target groups in the development of new digital public health interventions could be to apply user-centered approaches to the development process [28]. The aim here is to look at issues from various stakeholder perspectives and create new ideas in an interdisciplinary team to solve potential problems and challenges throughout the development of a digital public health intervention. Ideally, this approach also includes the target group (eg, for an app) to increase acceptance and use. Generally, participatory development processes are iterative and may be designed in various forms depending on the goal. In principle, the following four steps can shape the process: (1) concept generation and ideation; (2) prototype design and system development; (3) Evaluation; and (4) deployment, including various feedback loops (Figure 3). After an initial analysis of the user’s needs, the developers collect the criteria for functions and design. Then, they convert these recommendations into the functional specifications of a user-centered design. Using walkthroughs and usability testing, prototypes are tested and perfected before deployment, which helps to expose latent practical and interface design weaknesses. The developing team can achieve this by analyzing remotely collected data using automatic data transmission or video use. As usability testing is a pillar of the best practices for medical system architecture [28], production teams should also apply this to digital public health interventions’ development.

**Table 1:** The 9 different steps for including participant perspectives [6].

Participation and steps			Descriptions
**No participation**			
	Step 1: instrumentalization	1.1 The interests of the target group are not necessary 1.2 Production team makes decisions outside the target group 1.3 The interests of the decision-makers are the focus of attention 1.4 Target group members as decoration	
	Step 2: instruction	2.1 The situation of the target group is perceived 2.2 The problem is defined exclusively from the perspective of the decision-makers (professionals) 2.3 The opinion of the target group is not considered 2.4 Communication is direct	
**Precursors for participation**			
	Step 3: information	3.1 The decision-makers tell the target group what problems the group has and what help they need 3.2 Recommendation of various courses of action 3.3 Explanation and justification of the procedure of the decision-makers 3.4 The point of view of the target group is considered to increase the acceptance of the messages	
	Step 4: consultation	4.1 The decision-makers are interested in the view of the target group. 4.2 The members of the target group are listened to	
	Step 5: involvement	5.1 The decision-makers are advised by (selected persons from) the target group	
**Participation**			
	Step 6: co-determination	6.1 The decision-makers consult with the target group 6.2 Negotiations between target group representatives and decision-makers 6.3 The target group members have a say	
	Step 7: partial transfer of decision-making authority	7.1 A right of participation in the decision-making process 7.2 Decision-making authority is limited to certain aspects	
	Step 8: decision-making power	8.1 The target group itself determines all essential aspects 8.2 Partnership-based cooperation between all parties involved 8.3 Accompaniment or support of others	
	Step 9: self-organization	9.1 The responsibility for a measure or a project is entirely in the hands of the target group	

## Discussion

### Principal Findings

The aim of this viewpoint paper was to define digital public health interventions and provide an exemplary classification framework for digital public health interventions. Such an approach may help identify core areas of digital public health interventions, which in turn might be helpful during the development and evaluation phases of digital public health interventions. We argue that it is crucial to examine digital public health interventions from 3 different perspectives. The first one should be the WHO framework for EPHF [4]. This is important as it provides an overview of what kind of activities, which strengthen and maintain health at the population level, belong to public health as a discipline and, therefore, what a public health intervention may be. The second perspective focuses on the digital aspects of an intervention. A suitable framework is the *Evidence Standards Framework for Digital Health Technologies* by NICE, as it classifies digital interventions based on their functions and defines corresponding evidence standards [5]. Both frameworks combined enable us to categorize digital public health interventions according to the area of public health and the level of interaction between the user and the digital tool. The last perspective focuses on user involvement in the development of such interventions, as proposed by Wright [6]. This is of great importance, as studies suggest that the acceptance of target users increases with more involvement in the process of development, testing, and implementation. Therefore, acknowledging the 9 levels of user participation (and focusing on levels 6 to 9) may enable developers to create even more significant and meaningful digital public health interventions for their target group.

Our current approach relates to a single and, to the best of our knowledge, the only definition of digital public health. As it is natural for such definitions to evolve over time as the field evolves, our suggestion for a definition of digital public health interventions might also evolve, as one cannot talk about a definition for digital public health interventions without defining the borders of digital public health. Although the suggested EPHF in this perspective piece refers to a summary of the WHO, some readers of this paper might find it hard to apply it to their specific context. This may very much depend on the health care system in which the digital public health intervention is developed. Therefore, the EPHF listed in this study should not be seen as a final list of public health functions or classification frameworks but rather as examples of core functions and goals. Similarly, the NICE framework might not be applied directly in other countries with different health systems and contexts; however, it might provide a helpful starting point for identifying relevant frameworks for such systems or developing their own frameworks that focus on interaction and functional classifications. Possible steps for participation to include user perspectives and methods (eg, user stories) might differ depending on the format and content of a specific intervention. For example, one cannot expect an app that facilitates communication between physicians and patients to unfold its full potential when the development team does not consider both perspectives regarding design, functions, and content [29,30]. Some effects might be more visible on a public health scale than others, depending on the population’s size and the health system for which the intervention was initially developed. More importantly, digital public health interventions should display their effectiveness beyond the laboratory in the real world. They should do so by providing study results with high internal validity and results with high external validity. This well-known approach within empirical social research ensures that measurable effects transpire from the laboratory to the real world.

The following example aims to display the connections among the 3 analyzed frameworks and models. Since the beginning of the SARS-CoV-2 pandemic in early 2020, various countries have developed contact-tracing apps for monitoring and surveillance [31]. The primary function of such apps is to notify users after contact with someone who was (later) tested positive for the SARS-CoV-2 virus [32]. As previously mentioned, contact-tracing apps serve the horizontal EPHF of health information systems. Most countries set the bars for data security in contact-tracing apps high to improve users’ trust. Conversely, a high level of data protection prevents the collection and analysis of epidemiologically relevant data, making it more difficult to assess the effectiveness from a public health perspective. When developing a contact-tracing app, it is necessary to weigh the protection of privacy and the potential public health benefits against each other [33]. This constraint of data availability for public health (research) limits contact-tracing apps to evidence tiers 1 and 2 within the NICE framework for DHIs. Although simple monitoring (as level 2 demands) is possible, the apps do not aim to calculate the diagnoses needed for tier 3 (instead, recommendations such as different colors for warning levels in the German app).

As previously mentioned, participation in the development process is key to a successful intervention. Germany introduced the *Corona Warn App* in June 2020. The code was published as an open-source project in May 2020 on the GitHub coding platform [34]. According to the developers, this approach should allow interested target group users to assess the code for themselves and add suggestions to improve the app [34]. It is also possible to claim an interest in working on a specific part of the app’s code, which suggests a high level of involvement. Target group users are not just listened to but can also actively participate in the development process. However, although this approach offers a high level of participation for some members of the target group with a background or interest in coding, this approach excludes most other users because of their missing knowledge in information and communication technology. Furthermore, no clear information on the extent to which user groups were included in the actual development process is available. Despite the limited involvement of the target group, the app was downloaded 28.3 million times with 472.960 positive tests shared within the app by June 11, 2021, suggesting at least some success [35].

### Conclusions

This study aimed to provide the first definition and classification framework for digital public health interventions. Here, we suggest that digital public health, as a complex intervention, should be viewed from different perspectives. As such, our proposed classification is a combination of the elements of already existing models, specifically, the EPHF by the WHO, which provided us with an overview of the necessary core functions of public health that might also be addressed by digital means. The NICE framework gave us an overview of different areas for digital technologies and potential evaluation requirements. Both models together form a framework for describing digital public health interventions. However, without the inclusion of target groups in user-centered processes during the development, these interventions may lack efficiency and the acceptance of potential users. Therefore, we propose an established user-centered design process to be included in the development of digital public health interventions. Nevertheless, users of our definition and framework must check the validity of our criteria within their setting (eg, population structure, understanding of public health, and health care system). Taking the different strains of research that together might provide a better understanding of the term *digital public health intervention*, the first definition might be as follows:

A Digital Public Health Intervention addresses at least one essential Public Health function through digital means. Applying a framework for functional classification and stratification categorizes its interaction level with the user. The developmental process of a digital public health intervention includes the user perspective by applying participatory methods to support its effectiveness and implementation with the goal to achieve a population health impact.

The first step toward a potential intervention classification framework was developed based on this definition and its underlying frameworks (Multimedia Appendix 1). The aim of this framework is three-fold: (1) support the future reporting of digital public health intervention functions and effectiveness by providing a framework for classification, (2) identify future requirements (eg, for evaluation) of such interventions, and (3) support the implementation processes of digital public health interventions by linking implementation needs and characteristics with the classification framework (ie, a digital public health intervention addressing active monitoring in health care with high levels of user involvement might have other implementation needs than a digital public health intervention that addresses simple monitoring in health care with low levels of user involvement) [36]. We view a combination of all 3 models as a chance to set up a first definition and classification for digital public health interventions and hope that our approach will encourage the uptake and further development of our idea.

## Appendix

Multimedia Appendix 1 Digital public health intervention classification framework.

## Abbreviations

DHI: digital health intervention
EPHF: Essential Public Health Function
mHealth: mobile health
NICE: National Institute for Health and Care Excellence
WHO: World Health Organization
