# Supplementary material for: What are Digital Public Health Interventions? First Steps Toward a Definition and an Intervention Classification Framework
Source: J Med Internet Res. 2022 Jun 28;24(6):e31921. doi: 10.2196/31921 (PMC9277526; doi:10.2196/31921)
Supplement: Multimedia Appendix 1 [file jmir_v24i6e31921_app1.docx]

Multimedia Appendix 1: Digital Public Health Intervention Classification Framework

| **Functions and Fields for DiPH Interventions** | | | | | | | | | |
| --- | --- | --- | --- | --- | --- | --- | --- | --- | --- |
| **Digital**  **Function**  **PH Area** | System  Service | Information | Simple  Monitoring | Communication | Preventive Behavior Change | Treatment | Active Monitoring | Calculation | Diagnostic |
| Governance |  |  |  |  |  |  |  |  |  |
| Financing |  |  |  |  |  |  |  |  |  |
| Human  Resources |  |  |  |  |  |  |  |  |  |
| Health Information  Systems |  |  |  |  |  |  |  |  |  |
| Research |  |  |  |  |  |  |  |  |  |
| Social Participation & Health  Communication |  |  |  |  |  |  |  |  |  |
| Health  Protection |  |  |  |  |  |  |  |  |  |
| Health  Promotion |  |  |  |  |  |  |  |  |  |
| Disease  Prevention |  |  |  |  |  |  |  |  |  |
| Health Care |  |  |  |  |  |  |  |  |  |
| Preparedness for PH Emergencies |  |  |  |  |  |  |  |  |  |
| Other Area |  |  |  |  |  |  |  |  |  |

| **Level of Participation** | | | | | | | | | |
| --- | --- | --- | --- | --- | --- | --- | --- | --- | --- |
| **Level** | Instrumentalization | Instruction | Information | Consultation | Involvement | Code-  Termination | Partial Transfer of  decision-making  Authority | Decision-making Power | Self-  Organization |
| **Achieved** |  |  |  |  |  |  |  |  |  |

*Note:* PH = Public Health; DiPH = Digital Public Health
